# Supplementary material for: Dynamics of scene representations in the human brain revealed by magnetoencephalography and deep neural networks
Source: Neuroimage. Author manuscript; Available in PMC 2017 Aug 3. (PMC5542416; doi:10.1016/j.neuroimage.2016.03.063)
Supplement: 1 [file NIHMS882782-supplement-1.docx]

**SUPPLEMENTARY INFORMATION**

**Dynamics of scene representations in the human brain revealed by magnetoencephalography and deep neural networks**

Radoslaw Martin Cichy^1^, Aditya Khosla^1^, Dimitrios Pantazis^2^, Aude Oliva^1^

^1^ Computer Science and Artificial Intelligence Laboratory, MIT, Cambridge, MA, USA

^2^ McGovern Institute for Brain Research, MIT, Cambridge, MA, USA

# SUPPLEMENTARY FIGURES & CAPTIONS


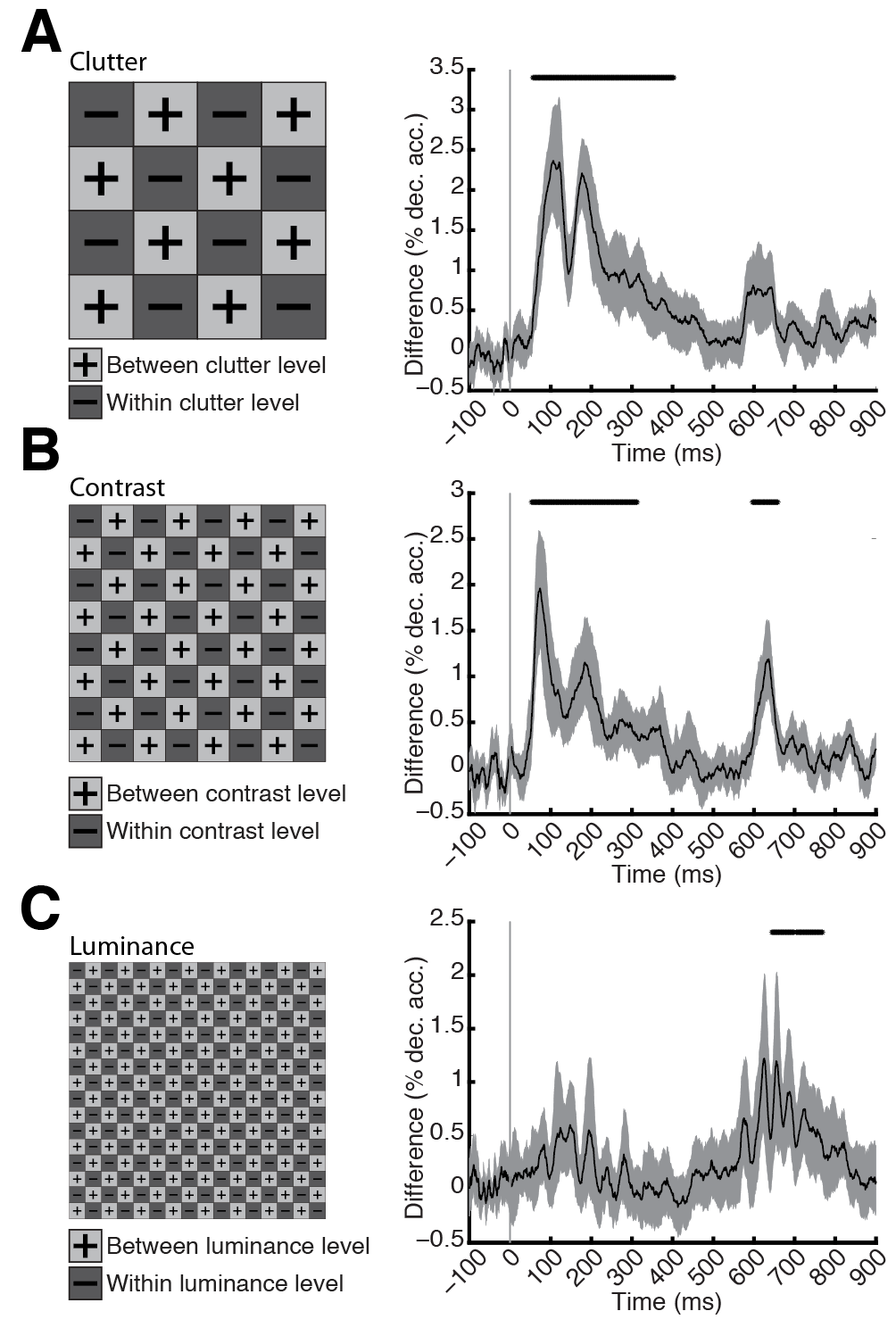


**Supplementary Figure 1: Time courses with which clutter, contrast, and luminance representations emerged in the brain.** The MEG decoding matrix was partitioned by **A)** clutter, **B)** contrast and **C)** luminance level (left column), and the mean within-subdivision decoding accuracies (dark gray, –) were subtracted from between-subdivision decoding accuracies (light gray, +) to estimate time-resolved representations (right column). Horizontal lines indicate significant time points (*n*=15, cluster-definition threshold *P* < 0.05, corrected significance level *P* < 0.05); gray shaded area indicates 95% confidence intervals determined by bootstrapping participants; gray vertical line indicates image onset.


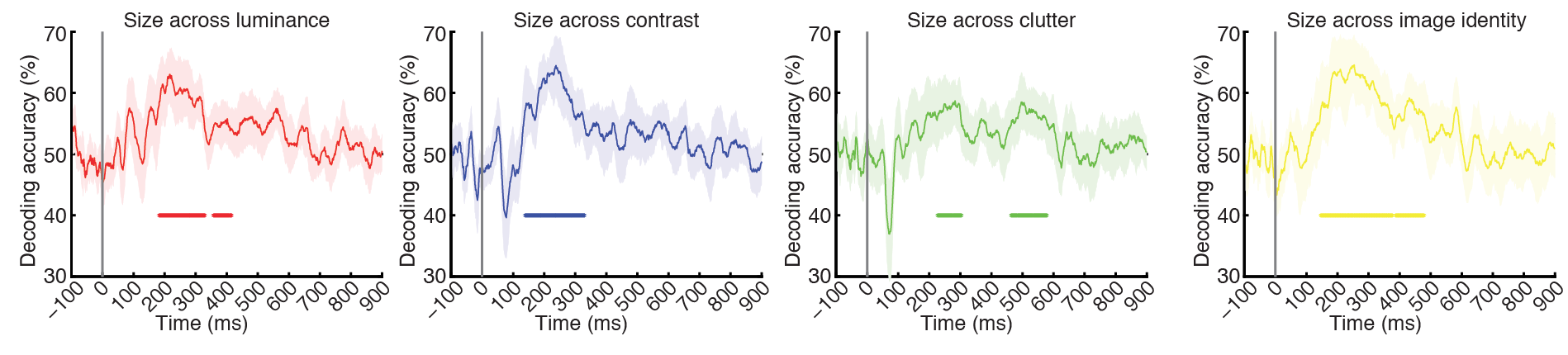


**Supplementary Figure 2: Results of cross-classification analysis plotted with 95% confidence intervals as determined by bootstrapping participants (colored shaded areas).** Horizontal lines indicate significant time points (*n* = 15, cluster-definition threshold *P* < 0.05, corrected significance level *P* < 0.05); gray vertical line indicates image onset.


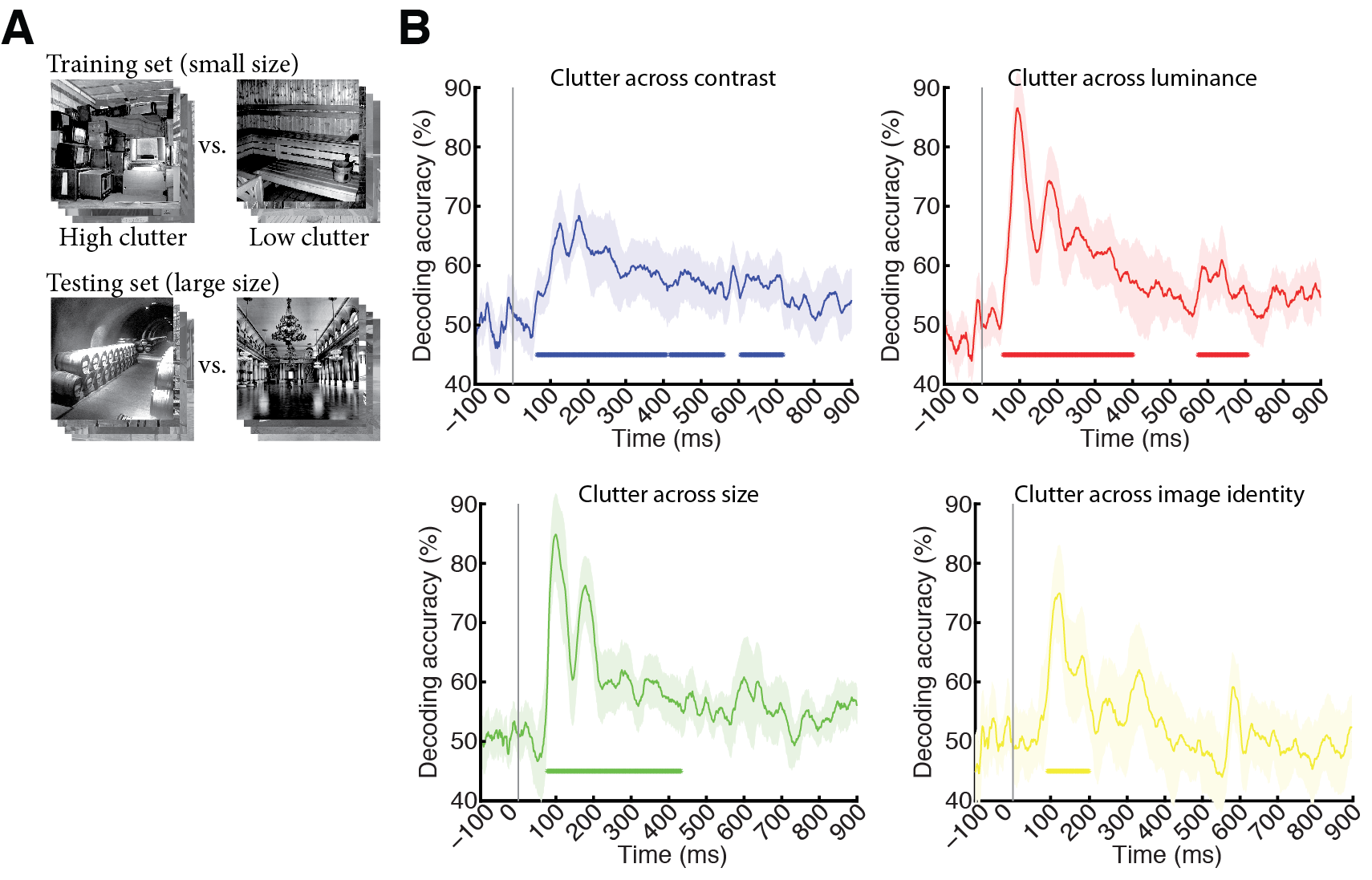


**Supplementary Figure 3. Scene clutter representations emerged with a distinct time course and were robust to changes in viewing conditions and other scene properties. A**) Cross-classification analysis, exemplified for cross-classification of scene clutter across scene size. **B)** Results of cross-classification analysis indicated tolerance of visual representations of scene clutter to changes in other scene and image properties (scene size, contrast, luminance and scene image identity). Horizontal lines indicate significant time points (*n*=15, cluster-definition threshold *P* < 0.05, corrected significance level *P* < 0.05); colored shaded areas indicate 95% confidence intervals determined by bootstrapping participants; gray vertical line indicates image onset.


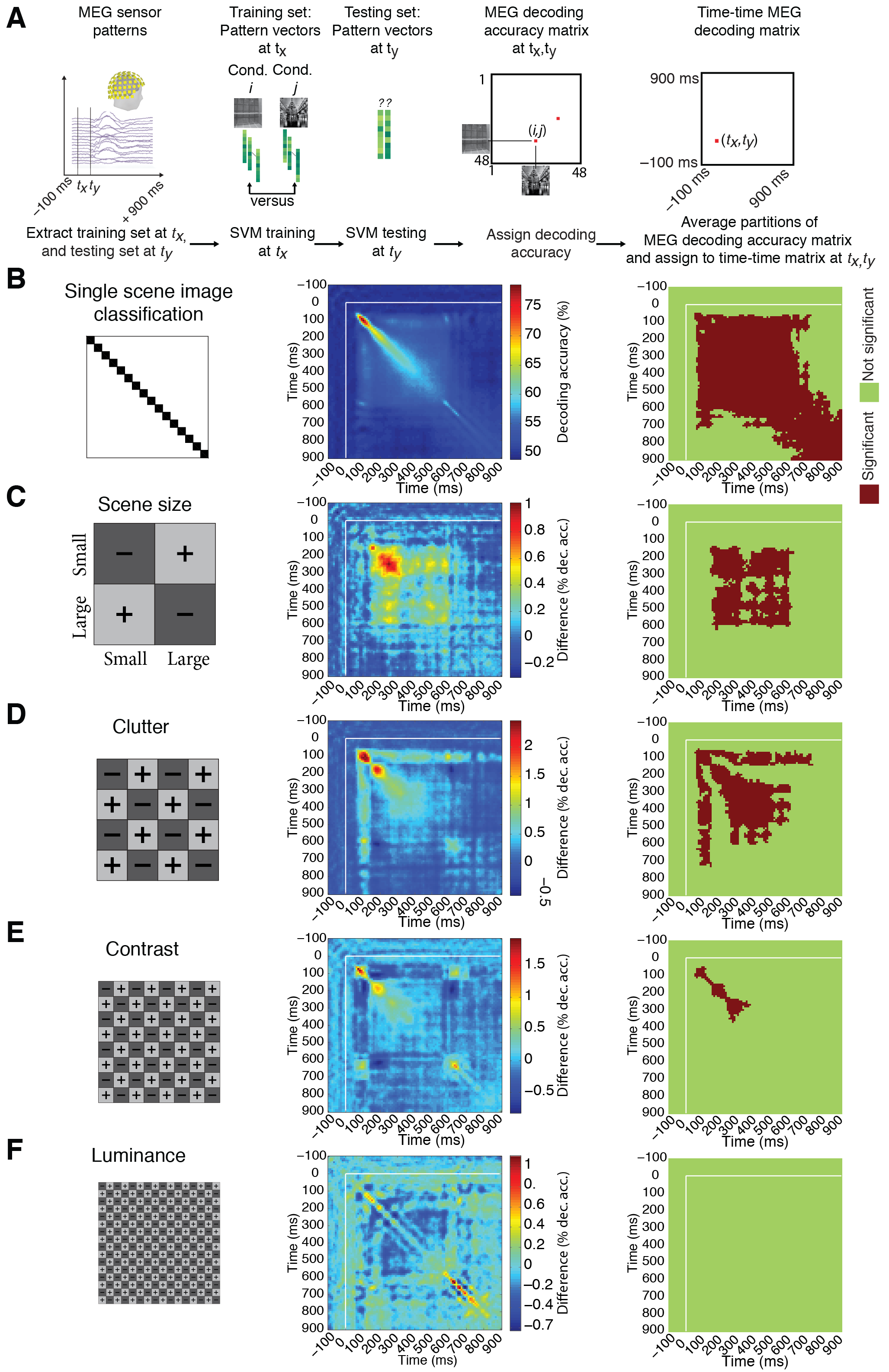


**Supplementary Figure 4. Persistent components in scene representations. A)** While early visual processes are highly dynamic, changing rapidly one transient representation to another, emerging scene representations critical for behavioral guidance may be persistent, lasting hundreds of milliseconds, to ensure subsequent access (Kriegeskorte and Kievit, 2013; Cichy et al., 2014; King and Dehaene, 2014). We reasoned that if visual representations persist over time, MEG signals should be similar across time as well. To evaluate, we trained a SVM classifier to distinguish scene images from MEG brain responses at time t_x_ and tested on data at other times t_y_. Repeating this procedure for all pairwise combinations of conditions and all time points produced a 4D condition × condition × time × time matrix. **B-F**) Averaging over or comparing by subtraction different parts of the first two dimensions (left column, same as in Figure 1C (scene image identity), Figure 2A (scene size) and Supplementary Figure 1 1) yielded 2D time-time decoding accuracy matrices (subject-averaged results in middle column; significant values in right column). Most cases exhibited strong transient components, as evidenced by higher decoding accuracies along the diagonal (t_x_ ≈ t_y_) than surrounding points in the time-time decoding matrices, with only exemption luminance. Persistent components had significant points outside the diagonal (t_x_ ≠ t_y_), and were evidenced for single scene image classification, scene size and scene clutter (B-D), but not for contrast or luminance (E, F). (*n*=15, sign-permutation tests, cluster-definition threshold *P* < 0.0005 for B and *P* < 0.05 for C-F, cluster threshold *P* < 0.05).


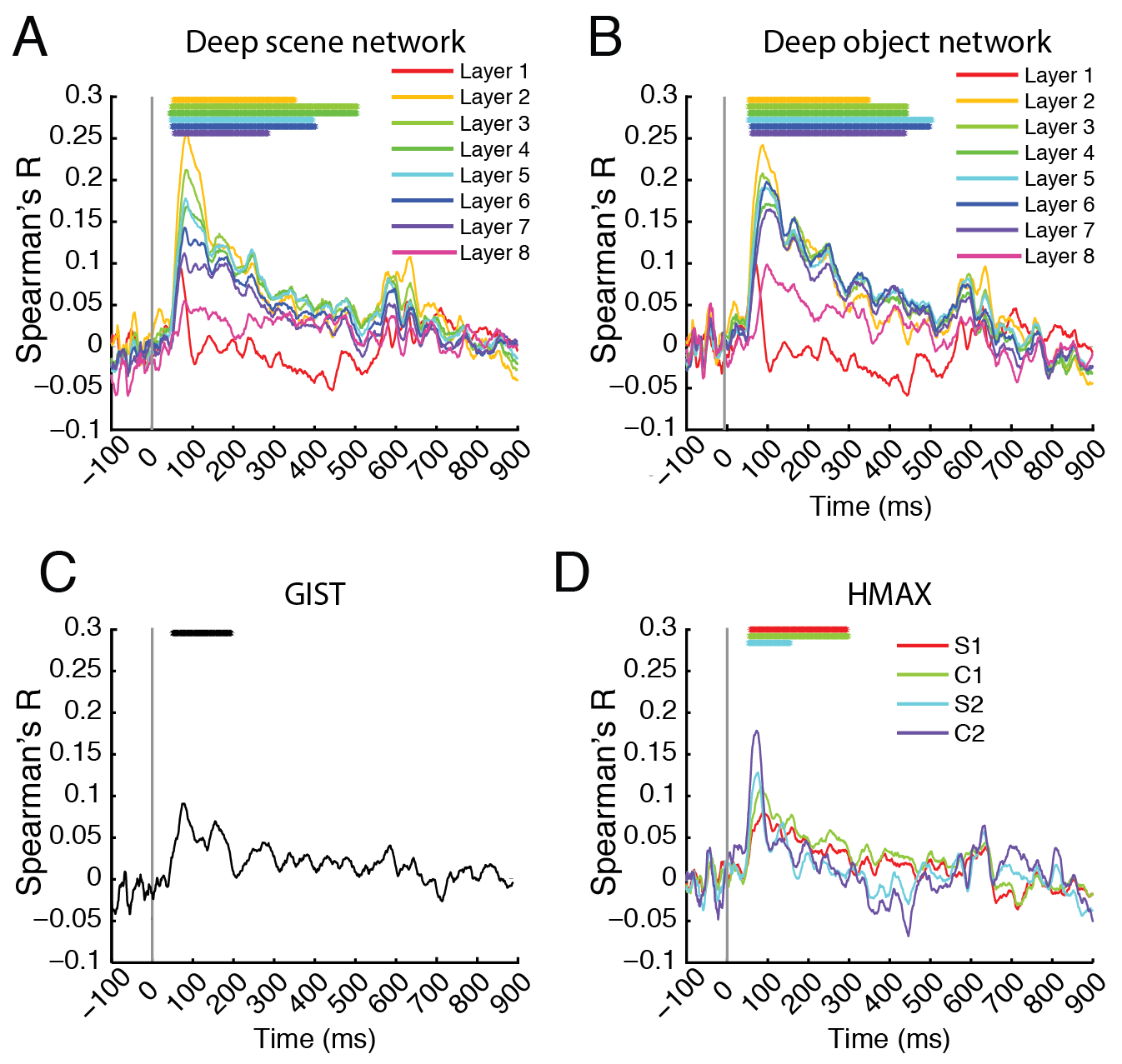


**Supplementary Figure 5. Layer-resolved prediction of emerging neural representations of single scene images by computational models.** **A)** Deep scene network, **B)** Deep object network, **C)** GIST, **D)** HMAX. Horizontal lines indicate significant time points (*n*=15, cluster-definition threshold *P* < 0.05, corrected significance level *P* < 0.05 divided by model-specific layer number); gray vertical line indicates image onset.


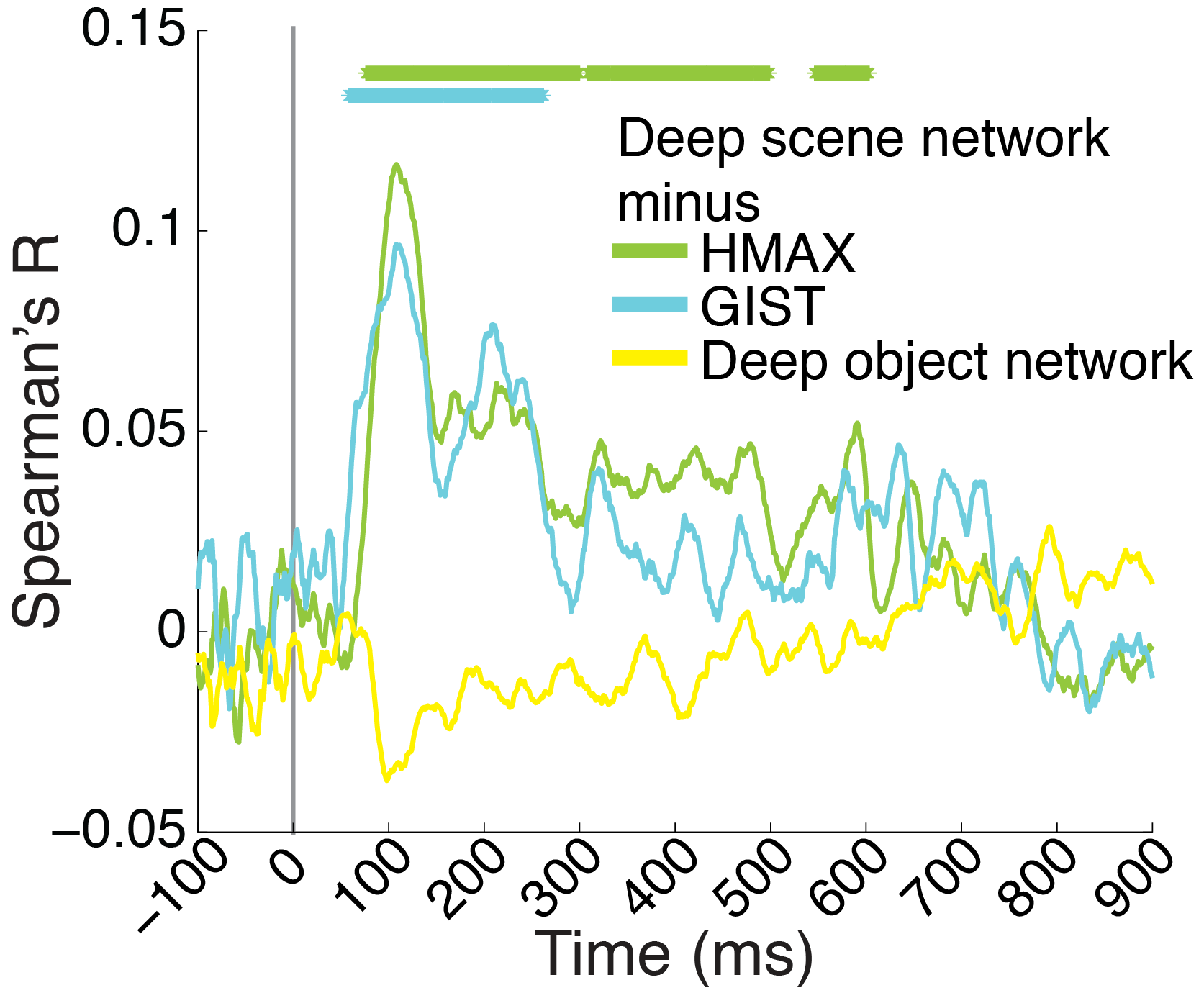


**Supplementary Figure 6. Comparison of the deep scene network against other computational models in predicting neural representations of single scene images.** To directly compare the time series in Figure 3D, we subtracted the time series of the HMAX, GIST, and deep object network from the time series of the deep scene network. While the deep scene and object networks did not differ significantly, the deep scene network predicted MEG better than HMAX and GIST. For details see Table 2b. Horizontal lines indicate significant time points (*n* = 15, cluster-definition threshold *P* < 0.05, corrected significance level *P* < 0.05); gray vertical line indicates image onset.


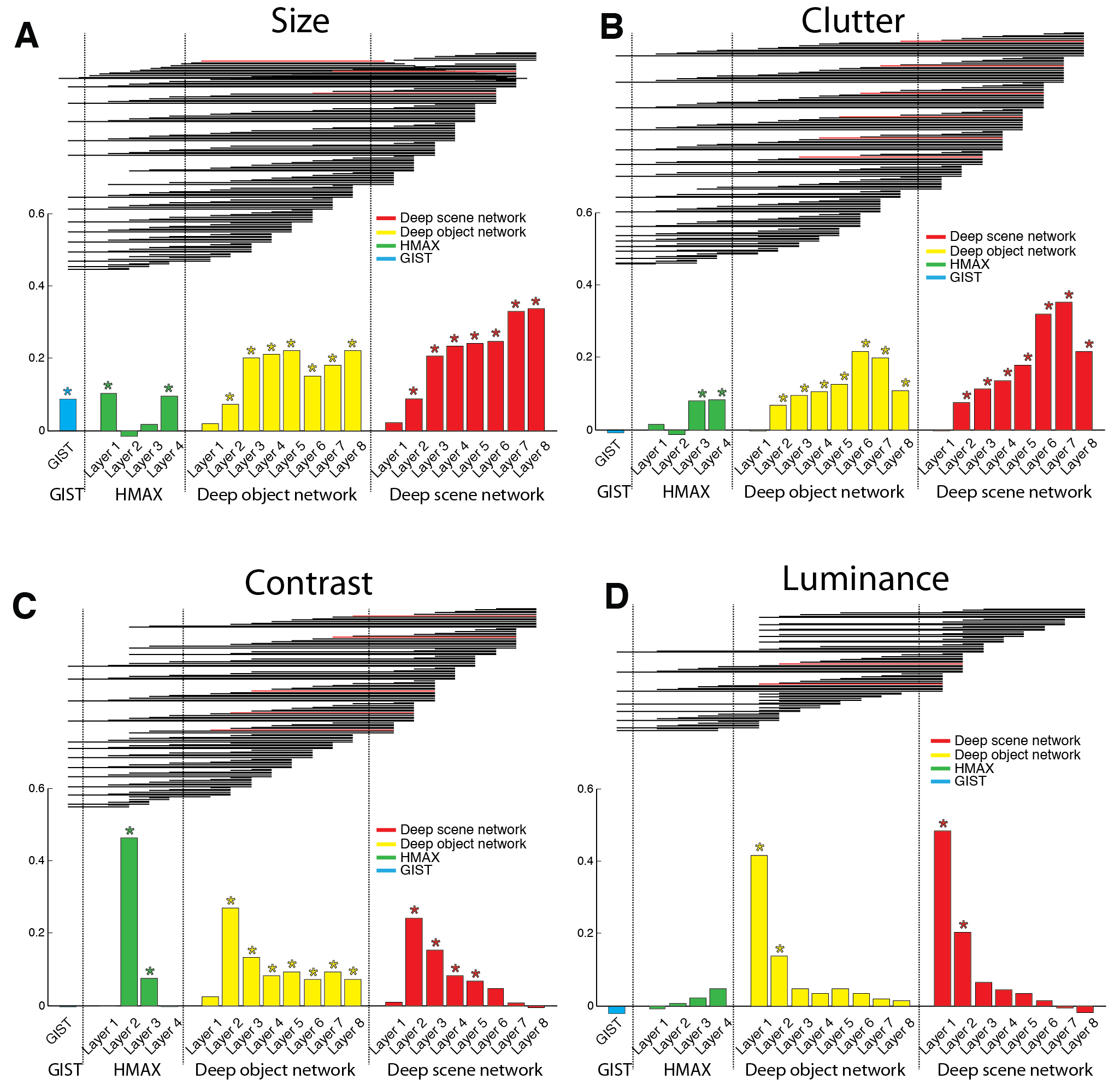


**Supplementary Figure 7. Complete layer-wise comparisons of scene representations in computational models of object and scene categorization.** Comparisons for **A)** scene size, **B)** clutter level, **C)** contrast level and **D)** luminance level. Bars indicate correlation between the computational model RDMs and explicit models of size, clutter, contrast, or luminance (e.g. the explicit size model in Figure 4E inset: an RDM with entries 0 for images of similar size, 1 for images of dissimilar size). While abstract scene properties emerged with increasing layer size in the deep scene and object networks progressively with increasing layer number, the low-level image properties contrast and luminance were progressively abstracted away. Lines above bars indicate significant differences across layers. Comparisons across equivalent layers of the deep scene and object networks are colored red. (*n* = 48, label permutation tests for statistical inference, *P* < 0.05, FDR-corrected for multiple comparisons for all layer-wise comparisons).


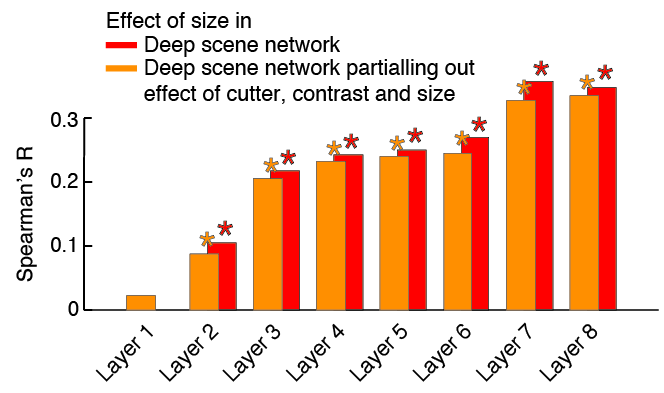


**Supplementary Figure 8. Representation of size effect in the deep scene network with vs. without partialling out the effect of other experimental factors (clutter, contrast and luminance).** To control whether the size effect observed in the deep scene network was explained by correlation with other experimental factors, we calculated the correlation between layer-specific RDMs and the size model before and after partialling out the model RDMs for clutter, contrast and luminance. We found that the size effect persisted in the model RDM in the partial correlation analysis, indicating that it is not explained by other factors. Stars above bars indicate statistical significance. (*n* = 48; label permutation tests for statistical inference, *P* < 0.05, FDR-corrected for multiple comparisons).


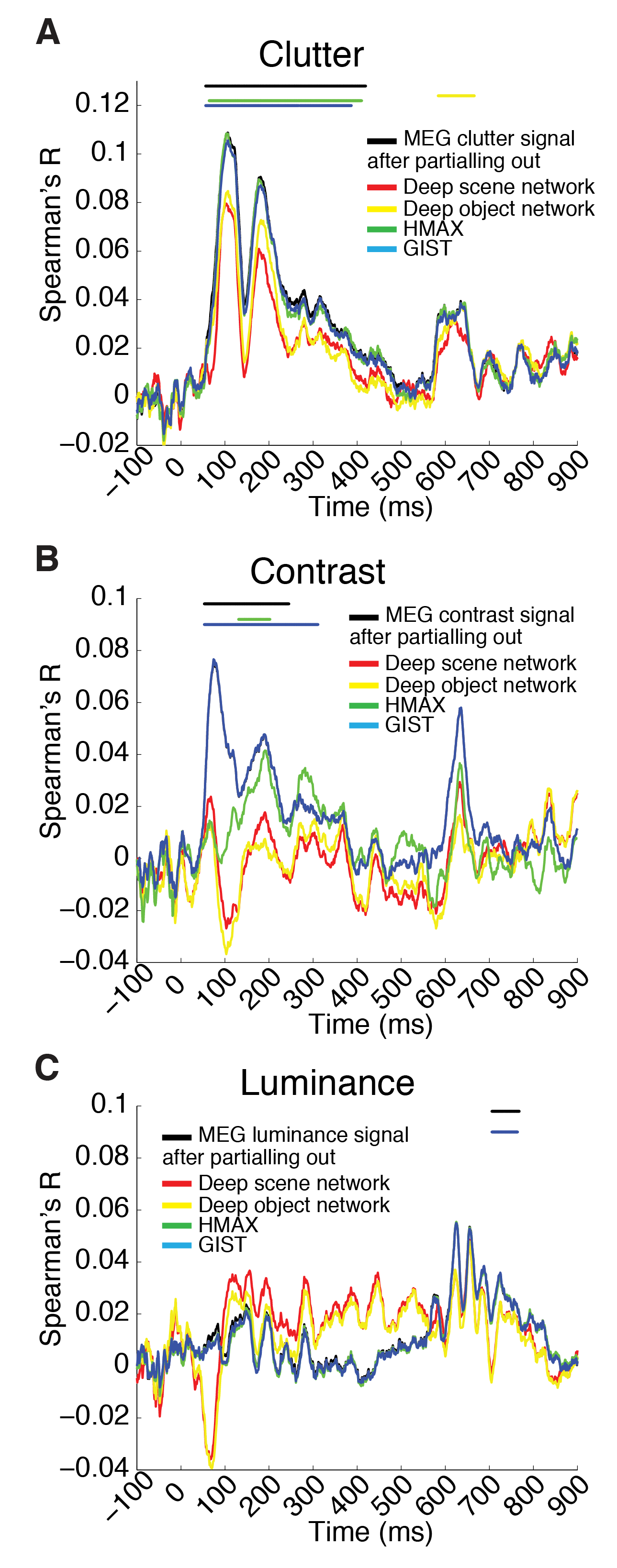


**Supplementary Figure 9. Neural representations of scene clutter, contrast, and luminance explained by computational models. A)** MEG representations of scene clutter, (termed MEG clutter signal) before (black) and after (color-coded by model) partialling out the effect of different computational models. **B-C)** same as **(A)** for contrast and luminance. Only partialling out the deep scene network abolished experimental effects for all factors. (*n* = 15; cluster-definition threshold *P* < 0.05, significance threshold *P* < 0.05 corrected for multiple comparisons by 5 for all panels).


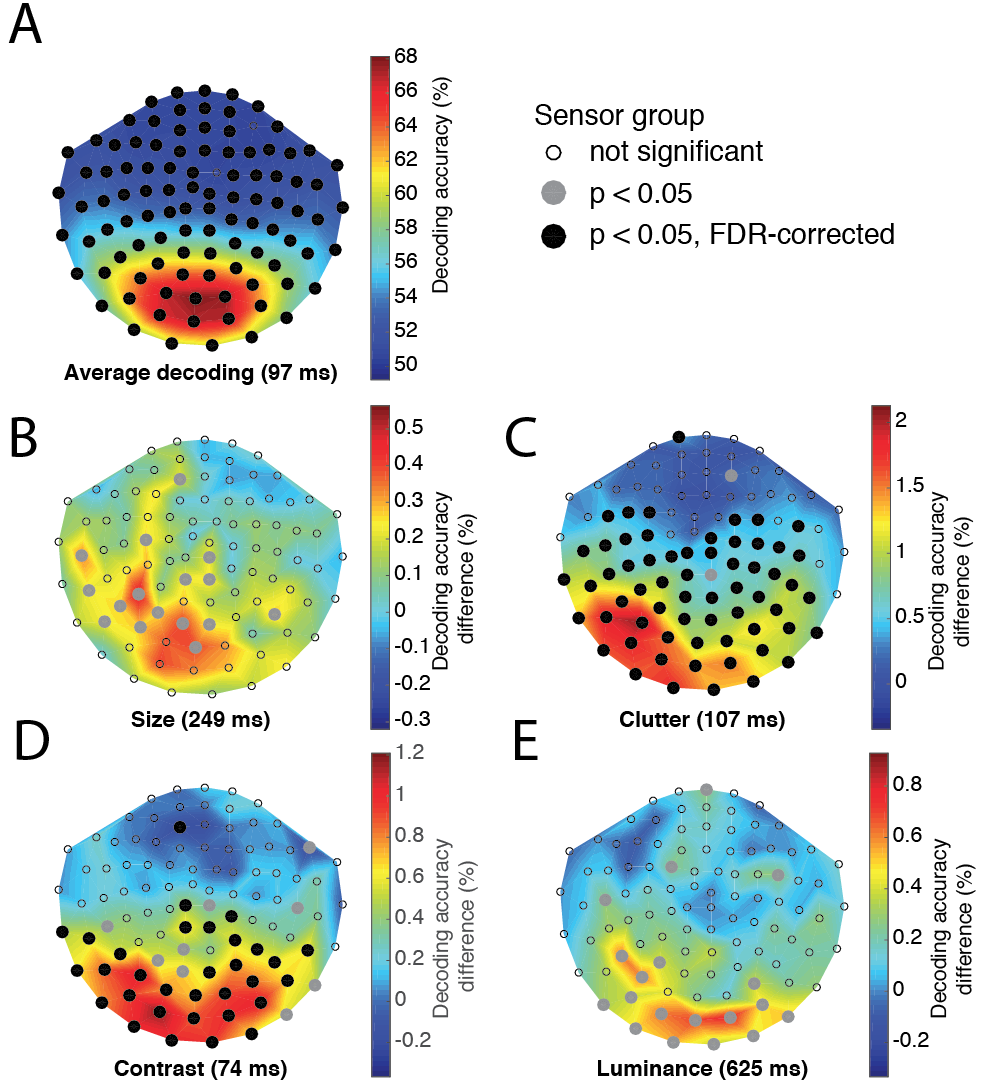


**Supplementary Figure 10. Sensor-wise decoding at the peak latency of observed experimental effects for A) average decoding, B) size, C) clutter, D) contrast and E) luminance.** To investigate which sensors contributed to observed experimental effects, instead of whole sensor analysis we conducted an equivalent decoding analysis for each MEG channel (306) separately at the peak latency of the effect in question. Decoding accuracy was averaged across sensors sharing the same position in the MEG helmet (102 triple sensor elements comprising two gradiometers and one magnetometer in the same location each; Elekta Triux MEG device), yielding decoding maps of 102 data points. Effect maps (averaged decoding accuracy in A, between- minus within-category averaged decoding accuracy difference in B-E) averaged across participants are shown in hot colors scale. For all cases, the MEG sensors showing highest experimental effects were positioned posterior in the helmet. This suggests that occipital and peri-occipital cortices are the sources of the observed effects when pooling information across sensors. The lack of strong decoding accuracy at frontal electrodes, i.e. close to the eyes, argues against eye movements as a determining factor. To assess significance, we conducted sign-permutation tests for each sensor (1,000 permutations). MEG sensors showing significant results at p < 0.05 are plotted in gray, and at p < 0.05 in black (FDR corrected for multiple comparisons for the 102 MEG sensor groups). For average decoding, nearly all electrodes showed significant effects. This is likely due to the smoothness of the MEG signals, though the decoding accuracy peak is clearly in posterior electrodes. For size, clutter, contrast and luminance, significant decoding effects were largely limited to posterior MEG sensors. In sum, cortical sources of observed effects are occipital and peri-occipital, rather than related to eye movements.


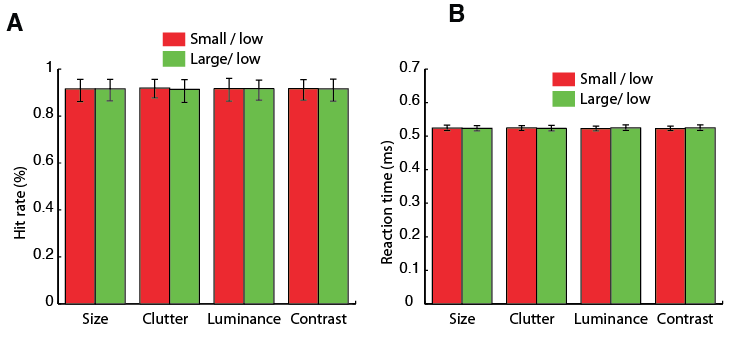


**Supplementary Figure 11. Behavioral analysis. A)** Hit rates and **B)** reaction times averaged across subjects (N=11) with 95% confidence intervals (bootstrapping pool of participants) for both factor levels ordered by each experimental factor. Reaction times (for hits) and hit rates were ordered by the preceding trial condition, averaged across runs, and then ordered according to level of the four different experimental conditions. Hit rates and reaction times were similar across factor levels for each factor. Statistical analysis (sign permutation tests, 1000 permutations) did not show significant differences in hit rates or reaction times between factor levels for any factor. For hit rates (size: *P =* 0.88; clutter *P =* 0.30; luminance *P =* 0.99 contrast *P =* 0.87), for reaction times (size: *P =* 061; clutter *P =* 0.39; luminance *P =* 0.09 contrast *P =* 0.15). This suggests that attentional load does not explain the observed differences in MEG signals between levels of experimental conditions.

# SUPPLEMENTARY MOVIE

**Supplementary Movie 1. MEG decoding accuracy matrix and multidimensional scaling solution in two dimensions.** The movie shows the MEG decoding accuracy matrices over time in 1 millisecond steps, accompanied with a two-dimensional scaling solution (criterion: metric stress). Experimental conditions (images) are plotted in the two dimensional space, color- and shape-coded to indicate scene size and clutter level.
